# Effectiveness, Acceptability, and Feasibility of Digital Health Interventions for LGBTIQ+ Young People: Systematic Review

**DOI:** 10.2196/20158

**Published:** 2020-12-03

**Authors:** Dylan Gilbey, Helen Morgan, Ashleigh Lin, Yael Perry

**Affiliations:** 1 Telethon Kids Institute Perth Australia; 2 School of Psychological Science The University of Western Australia Perth Australia; 3 Discipline of Psychology College of Science, Health, Engineering and Education Murdoch University Perth Australia; 4 Centre for Child Health Research The University of Western Australia Perth Australia

**Keywords:** systematic review, mental health, physical health, sexual health, youth, sexuality, gender, mobile phones

## Abstract

**Background:**

Young people (aged 12-25 years) with diverse sexuality, gender, or bodily characteristics, such as those who identify as lesbian, gay, bisexual, transgender, intersex, or queer (LGBTIQ+), are at substantially greater risk of a range of mental, physical, and sexual health difficulties compared with their peers. Digital health interventions have been identified as a potential way to reduce these health disparities.

**Objective:**

This review aims to summarize the characteristics of existing evidence-based digital health interventions for LGBTIQ+ young people and to describe the evidence for their effectiveness, acceptability, and feasibility.

**Methods:**

A systematic literature search was conducted using internet databases and gray literature sources, and the results were screened for inclusion. The included studies were synthesized qualitatively.

**Results:**

The search identified 38 studies of 24 unique interventions seeking to address mental, physical, or sexual health–related concerns in LGBTIQ+ young people. Substantially more evidence-based interventions existed for gay and bisexual men than for any other population group, and there were more interventions related to risk reduction of sexually transmitted infections than to any other health concern. There was some evidence for the effectiveness, feasibility, and acceptability of these interventions overall; however, the quality of evidence is often lacking.

**Conclusions:**

There is sufficient evidence to suggest that targeted digital health interventions are an important focus for future research aimed at addressing health difficulties in LGBTIQ+ young people. Additional digital health interventions are needed for a wider range of health difficulties, particularly in terms of mental and physical health concerns, as well as more targeted interventions for same gender–attracted women, trans and gender-diverse people, and people with intersex variations.

**Trial Registration:**

PROSPERO International Prospective Register of Systematic Reviews CRD42020128164; https://www.crd.york.ac.uk/prospero/display_record.php?RecordID=128164

## Introduction

Young people who are lesbian, gay, bisexual, transgender, intersex, queer and other people of diverse sexuality, gender, or bodily characteristics (LGBTIQ+) are known to experience a range of disparities in health outcomes compared with their peers [1]. These include higher rates of mental health difficulties, such as depression and suicidality [2-4]; physical and sexual health problems, such as the incidence of HIV [5]; cigarette and alcohol use [6-8], obesity [9], and teen pregnancy [10]. Later in adulthood, the confluence of these health issues conveys further risk for cancer [11] and cardiovascular disease diagnoses [12-14]. The burden of disease that these disparities carry is a public health issue that urgently requires safe, effective, and early intervention.

These health disparities are compounded by barriers that negatively impact the ability of LGBTIQ+ young people to access health services that are safe and adequately meet their needs. Young people in the general population face many barriers to help seeking, including inadequate resources and lack of accessibility, desire for self-reliance, and anticipated stigma for reporting certain health difficulties such as mental illnesses or HIV [15,16]. LGBTIQ+ young people face a range of additional difficulties, such as low parental support, which can lead to homelessness [17-19], and unemployment due to discrimination [20,21], which may intensify these help-seeking barriers [22]. This group also faces unique help-seeking difficulties, such as anticipated and experienced stigma surrounding their identities [22-24], concerns about disclosure or their compromised confidentiality [24], and low perceived confidence in the ability of service providers to deliver LGBTIQ+ supportive care [23-26]. These problems may be particularly pronounced among queer and trans and gender-diverse young people [25,27]. The factors impeding health care access and use may significantly contribute to the maintenance of health disparities in LGBTIQ+ young people [28,29]. Accordingly, removing these obstacles is an important step toward improving health in this population.

Digital health interventions, such as those delivered via computers, websites, smartphones, or tablets, have been identified as an important potential avenue to improve health care access and use among young people in this group [30-33]. Accessing support digitally allows young people to bypass many of the aforementioned barriers. Anonymity facilitates private access to support and minimizes stigma [33]. In addition, digital health interventions confer further benefits beyond traditional clinical environments, being available without travel, accessible at all hours, and having no waitlists [34]. Self-guided digital health interventions are also cost- and resource-effective to access and disseminate, requiring less direct input from clinicians [34], giving consumers greater control and empowerment regarding their own health needs [35]. These considerations are especially pertinent for widening the support available to populations that are restricted from accessing traditional health care services [36]. However, digital health interventions are also commonly reported to have very high rates of attrition and disengagement [37,38], with up to 60% to 80% of users discontinuing their use [39-41], and the quality of evidence supporting the effectiveness of digital health interventions is also frequently lacking [42-45]. Along with the limited availability of many evidence-based digital health interventions beyond the research context, these issues call into question the real-world utility of these interventions despite their proposed theoretical benefits.

LGBTIQ+ young people are adept and frequent users of digital technologies [33,46,47]; however, research indicates that technology use can present several challenges. Evidence suggests that the internet (including social media and online communities in particular) can be harmful in this population (as well as young people more broadly) [31] due to the potential for toxic interactions and exposure to distressing content [48], such as the normalization of self-harm and suicidal behaviors [49]. However, an array of benefits associated with technology use has also been documented in LGBTIQ+ young people. The internet allows LGBTIQ+ young people to explore their identities in an anonymous and safe way, seek out peers who share their identities, and come out to others in a low-risk environment [47]. The internet also facilitates an important component of the sexual development of LGBTIQ+ young people, enabling the exploration of same-gender attraction for some and seeking out romantic or sexual partners [50]. LGBTIQ+ young people may also already use the internet to access resources that are relevant and safe for them [47], suggesting that digital health interventions targeting this group may be useful. The concept of digital delivery of interventions is generally acceptable to this group, and this is particularly true when they are specifically targeted with LGBTIQ+ themes [32]. Many existing digital health interventions are not specifically applicable to LGBTIQ+ people [51]; however, untailored interventions may exacerbate feelings of alienation [31,52,53]. Given these factors, the development and evaluation of targeted digital health interventions for LGBTIQ+ young people may represent an opportunity to improve the delivery of health care to this group, should the benefits outweigh the known challenges associated with digital health care delivery discussed above.

In response, there has been a rapid increase in the number of such interventions over the past decade. To date, however, there has not been a comprehensive review summarizing the scope and use of digital health interventions that currently exist for this population. Knight et al [54] recently reviewed web-based interventions for HIV or sexually transmitted infection (STI) risk reduction in young men who have sex with men (MSM); however, this review did not capture digital health interventions that are delivered through other digital modalities (eg, mobile apps), those that address other health issues, or those that target women, gender-diverse individuals, or individuals with intersex variations. There have also been several recent reviews focused on the mental health of LGBTIQ+ young people [55,56] and adults [57], which have referenced a combined total of 4 digital interventions for this population. However, these reviews were not explicitly focused on the use of digital technology nor did they consider interventions designed to improve a broad range of health outcomes in this population.

A more extensive summary of this rapidly growing field of research will assist in identifying gaps in the development of interventions and determining the overall evidence for their use across the full diversity of the young LGBTIQ+ community. Therefore, this review aims to answer the following questions: (1) What are the characteristics of evidence-based digital health interventions for improving mental, physical, and sexual health outcomes in LGBTIQ+ young people? (2) Are targeted digital health interventions effective at improving health outcomes in LGBTIQ+ young people? (3) Are targeted digital health interventions acceptable and feasible for LGBTIQ+ young people?

## Methods

### Protocol and Registration

The protocol for this review was registered using PROSPERO (Prospective Register of Systematic Reviews; ID CRD42020128164) in accordance with PRISMA (Preferred Reporting Items for Systematic Reviews and Meta-Analyses) recommendations [58].

### Eligibility Criteria

#### Types of Participants

The population of interest was LGBTIQ+ young people. The LGBTIQ+ term was used in its broadest sense to capture young people of diverse sexuality (including but not limited to those that identify as gay, lesbian, bisexual, or pansexual), diverse gender (including but not limited to those who identify as trans or nonbinary), with diverse sex characteristics (including but not limited to people with intersex variations), or people falling across any combination of these categories. The search strategy (below) was designed to be as inclusive as possible of the wide variety of identities that LGBTIQ+ people may hold including, for example, people who fall within these aspects of diversity without explicitly identifying as such (eg, MSM). *Young people* was defined as being primarily people between the ages of 12 and 25 years; the mean age of study participants was required to fall within this range for a study to be eligible to be included in the review. In addition, studies were required to have intentionally and specifically recruited young people.

#### Types of Intervention

The review focused on interventions designed to effect change through predominantly digital means (eg, using a computer, website, tablet, or smartphone). To be included, interventions were required to be targeted or intended to specifically effect change in health outcomes in LGBTIQ+ people. Interventions delivered via telephone with no technological function or an implantable device that is remotely monitored were excluded. Interventions were also required to have minimal human guidance in the intervention itself if present at all. Specifically, the action, process of intervening, or behavior change techniques must have been delivered by the digital technology itself not a health professional working over a digital medium. This criterion was implemented because, in interventions that blend digital and human support, the impact of the intervention cannot be meaningfully attributed to the digital component alone [59].

The judgment of the level of human guidance was made by considering the ratio of clinicians or staff to users, and the centrality of the human guidance to effecting change in the health outcome, which itself relied on factors such as the ratio of guided versus unguided time during the intervention. No hard limits on these factors were set prior to conducting the review, as making this judgment required the full context of the intervention to be considered holistically. A judgment about the duration of the human guidance, for example, could not meaningfully be made without consideration of the purpose of that period of guidance and how it fits into the goals and process of the intervention as a whole. What was counted as *minimal human guidance* was therefore determined on a case-by-case basis requiring consensus from the reviewers.

#### Types of Studies

To be included, studies should have conducted an evaluation of a specific intervention as described above. Evaluation in some form was required in keeping with standards of evidence-based practice. The term *evaluation* was inclusive of examination of efficacy, effectiveness, acceptability, or feasibility, with a minimum of any one form of evaluation required for inclusion. All quantitative, qualitative, or mixed methods studies were eligible for inclusion. Comparators or control groups were not necessary for inclusion in the review. Protocols describing an intervention without any evaluation were not included in the review; however, when protocols were identified, steps were taken to determine if the corresponding data were publicly available. Studies evaluating the concept of digital health interventions generally or studies describing the initial development of an intervention were also ineligible. Studies evaluating digital health interventions not specifically designed for LGBTIQ+ young people were also excluded, even if conducted with an LGBTIQ+ sample.

No specification was made for the location of the study; however, studies were required to be published in the English language. With the aim of reducing the risk of publication bias [60], gray literature was considered eligible and studies were not required to be peer reviewed to be included in the review. The search was restricted to studies published after January 1, 2000. This cut-off was selected because of the types of interventions eligible for this review, only web-based interventions may have existed at this time, and the likelihood of such an intervention existing specifically tailored for a select, marginalized group was deemed to be extremely low. Scoping searches conducted before deciding on this cut-off did not determine any evidence of existing interventions contrary to this conclusion.

#### Types of Outcomes

The review was designed to capture interventions seeking to improve health outcomes or to prevent negative health outcomes. This was inclusive of mental health outcomes (eg, symptoms or diagnoses of mental disorders, well-being, distress), physical health outcomes (eg, smoking, weight loss), or sexual health outcomes (eg, pre-exposure prophylaxis [PrEP] adherence, condom use). Any outcome reasonably perceived to represent some aspect of health and well-being was considered relevant to the review. For studies evaluating efficacy, changes must have been reported in measures of at least one of these outcomes. For studies evaluating acceptability or feasibility, at least one index of these factors (eg, surveys of participant experiences, adherence, or attrition rates) must have been reported.

### Search Strategy

Internet databases such as PsycINFO (Ovid) and MEDLINE (Ovid) were systematically searched on August 13, 2019, and potentially relevant peer-reviewed publications were extracted. These searches were conducted using a combination of subject headings and keywords corresponding to the following themes: LGBTIQ+, Youth/Young People (aged 12-25 years), Mental Health, Physical Health, Sexual Health, Digital and Intervention. The search terms for LGBTIQ+, Youth/Young People, and Mental Health themes were adapted from those previously reported by Gilbey et al [61] and Lee et al [62]. The search terms for the other themes were devised from a broad initial scoping search of relevant articles to identify key terms and phrases. The search strategy for PsycINFO (Ovid) is presented in Multimedia Appendix 1.

Additional searches were made using Scopus, ProQuest Dissertations, Google, Google Scholar, OpenGrey, WorldCat, ClinicalTrials.gov, and JMIR Publications, during July and August 2019. Each of these searches were conducted with several simple keyword searches (eg, *LGBTQ*, *transgender*) as the relatively low number of relevant articles available in each source made a more comprehensive, and therefore restrictive, search process unnecessary. The reference lists of several other related reviews and key articles on the subject were also hand-searched for potentially relevant articles during July 2019. Google Scholar alerts were monitored for any additional articles published until April 2020. The searches of PsycINFO (Ovid) and MEDLINE (Ovid) were repeated on March 25, 2020, and articles published since August 2019 were manually searched for any newly published studies.

### Screening

The titles and abstracts of the articles identified by the search were screened for relevance by the lead author (DG), removing articles with no clear relevance to the topic of the review. Two authors (DG and HM) then screened the full-text of the remaining articles independently, with differences in opinion resolved in discussion with a third author (YP) in which full agreement was sought.

### Data Extraction

The following data items were extracted from eligible studies: author(s), year, participant age (mean and range), description of sample (eg, LGBTIQ+ status), sample size, study design, study setting, intervention type, content and delivery, digital platform, control group type (if relevant), degree of human guidance in the intervention, health outcome(s), acceptability outcome(s), and feasibility outcome(s). A second reviewer (HM) cross-checked these data.

### Critical Appraisal

Following data extraction, studies were evaluated using the Mixed Methods Appraisal Tool (MMAT) [63] by 2 reviewers (DG and HM). The MMAT is designed for the assessment of methodological quality of studies with a range of designs (qualitative, quantitative, and mixed methods), such as those reported herein. On the basis of the recommendations of the authors of the tool, quantitative quality scores were not derived; instead, the results of the appraisal are discussed qualitatively.

### Synthesis of Results

Owing to the wide array of interventions, targets of intervention, intervention modality, and health outcomes measured, it was anticipated that a quantitative synthesis (of those studies reporting quantitative data) would be neither feasible nor informative. Therefore, the results of the studies were synthesized qualitatively.

## Results

### Study Selection

**Figure 1:**
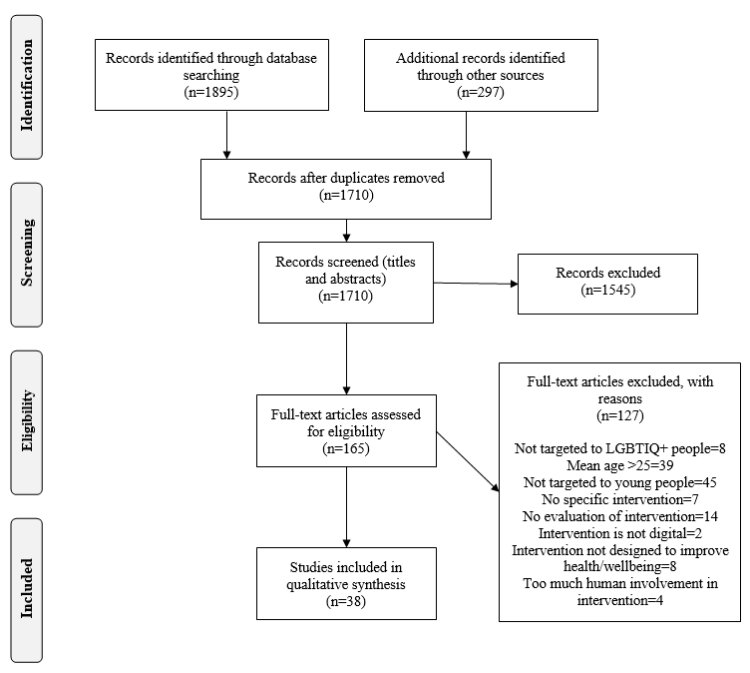
Study flowchart. LGBTIQ+: lesbian, gay, bisexual, transgender, intersex, queer.

The search and screening process is displayed in a PRISMA flow chart in Figure 1. A total of 2192 studies were identified in the search. Following title and abstract screening, 165 studies were retained for full-text screening. The final number of studies retained for the review following full-text screening was 38.

### Study Characteristics

The 38 studies captured the results of studies examining 24 unique interventions conducted across 3 countries (the United States, the United Kingdom, and New Zealand). Of these, 5 targeted primarily mental health–related issues, one targeted primarily physical health–related concerns, one targeted primarily sexual health and well-being, and 17 targeted risk reduction or management of STIs. A total of 22 interventions focused specifically on young people who are attracted to the same gender (referred to with a variety of terms, eg, sexual minority, lesbian/gay/bisexual/queer people, MSM), of which 19 interventions were described as being focused on young men (eg, gay/bisexual men, MSM). Several studies that described their target audience as MSM also included trans women under this descriptor. One study targeted people who identify as lesbian, gay, bisexual, transgender and queer (LGBTQ) generally [64] and one intervention targeted transgender men and nonbinary people specifically [65]. A total of 3 interventions specifically targeted young LGBTQ+ people of color, although several other interventions were also conducted with predominantly non-White participants. No studies were identified that sought to improve health in young people with intersex variations, and no studies were identified that sought to improve health in young women alone. The characteristics of the included studies are reported in Table 1. Because of the length of the table, the characteristics of the included studies addressing sexually transmitted infection risk reduction and management are reported separately in Multimedia Appendix 2 [66-91]. Brief summaries of each intervention as well as their core findings are also provided in Multimedia Appendix 3 [64-100].

**Table 1:** Summary of digital mental, physical and sexual health interventions for lesbian, gay, bisexual, transgender, intersex or queer young people.

Intervention category and name			Primary health outcome		Study		Participant mean age (range; intervention condition)		Description of sample (eg, LGBTIQ^a^ status/identification)		Sample size		Study design		Study setting		Intervention type		Digital platform		Primary measured constructs and effects
**Drug abuse prevention**																					
	Unnamed intervention	Drug abuse		Schwinn et al [92]		16.1 (15-16)		Sexual minority youth. Same-sex attracted=90, both-sex attracted=116, opposite-sex attracted=14, not sure= 13		236		Quantitative, RCT^b^ and follow-up, efficacy		United States		Interactive skill-building sessions		Computer via web		Alcohol use –^c^ Marijuana use – Cigarette use – Peer drug use ↓^d^ Other drug use ↓	
**Smoking cessation**																					
	Put It Out Project	Smoking cessation		Vogel et al [98]		19.7 (18-25)		Sexual and gender minority young adults. Gay/lesbian=6, Bisexual=15, Queer=2, Pansexual=8, nonbinary=10, Trans=2		27		Mixed methods, acceptability and feasibility		United States		Social media (Facebook)		Web		N/A^e^	
		Smoking cessation		Vogel et al [99]		21.4 (18-25)		Sexual and gender minority young adults. Gay=29, lesbian=30, bisexual/pansexual=93, other=13		165		Quantitative, RCT and follow-up, efficacy, and acceptability		N/A		Social media (Facebook)		Web		Number of cigarettes smoked weekly ↓ Self-reported smoking abstinence ↑^f^ Biochemically verified smoking abstinence ↑	
**Internalizing disorder prevention/management**																					
	Rainbow SPARX	Internalizing symptoms (depression and anxiety)		Lucassen [93]; thesis, studies two and three described in published articles below		N/A^e^		N/A		N/A		N/A		N/A		N/A		N/A		N/A	
		Internalizing symptoms (depression and anxiety)		Lucassen et al [94]		16.5 (13-19)		Sexual minority youth		21		Quantitative, uncontrolled pilot, acceptability and feasibility testing		New Zealand		Serious game		Computer via CD		Depressive symptoms ↓ Anxiety symptoms ↓	
		Internalizing symptoms (depression and anxiety)		Lucassen et al [94]		16.4 (13-19)		Sexual minority youth		25		Qualitative, acceptability testing		New Zealand		Serious game		Computer via CD		N/A	
		Internalizing symptoms (depression and anxiety)		Lucassen et al [53]		17.9 (15-22)		LGBT+ youth and health professionals		21 youth and 6 professionals		Qualitative, acceptability testing		United Kingdom		Serious game		Computer via CD		N/A	
	TODAY!	Internalizing symptoms (depression and anxiety)		Fleming et al [96]		19.0 (18-20)		Young sexual minority men. Gay=9		9		Qualitative, usability testing		United States		Mobile app		Mobile phone		N/A	
**Nonspecific mental health interventions**																					
	Unnamed intervention	Psychological distress		Pachankis and Goldfried [97]		20.2 (Range not provided)		Gay male college students		77		Quantitative, RCT and follow-up, efficacy		United States		Expressive writing		PC		Depressive symptoms – Psychological well-being –	
	QueerViBE	Psychological well-being		Martin [65]		18.0 (15-21)		Young trans men and nonbinary people. Trans male=89, nonbinary=50, questioning=5, other=12		156		Mixed methods, RCT, interviews, acceptability and efficacy		United Kingdom		YouTube videos		Web		Psychological distress ↓	
**Sexual health and wellbeing**																					
	Queer Sex Ed	Sexual health		Mustanski et al [64]		17.9 (16-20)		LGBT young people. Gay/lesbian=142, bisexual=31, queer=27, unsure/questioning=2, transgender=14		202		Mixed methods, one-arm pilot, acceptability, feasibility and efficacy		United States		Web-based curriculum		Computer via web		Sexual functioning ↑ HIV knowledge ↑ STD knowledge^g^ ↑ Contraceptive methods knowledge ↑ HIV testing location awareness ↑	

^a^LGBTIQ: lesbian, gay, bisexual, transgender, intersex, or queer.

^b^RCT: randomized controlled trial.

^c^–: No change.

^d^↓: Significant decrease.

^e^N/A: not applicable.

^f^↑: Significant increase.

^g^STD: sexually transmitted disease.

### Risk of Bias in Individual Studies

#### Mixed Methods Studies

Mixed methods designs were used well among the included studies overall, with most meeting all of the criteria in the MMAT for such designs. This is not to say that these studies did not have methodological flaws, as the MMAT mixed methods subsection does not generally consider the quality of the individual qualitative and quantitative components but rather their intersection and integration. These components of the studies were also, therefore, considered individually and are included among those described below.

#### Quantitative Studies

The majority of included quantitative trial studies were described as pilot or feasibility studies (17 out of 27), and their methodological quality was lacking in many cases. In total, 15 of the 27 trials were randomized trials, of which 4 did not report appropriate randomization procedures, 12 did not report blinding procedures, and participant adherence to the intervention was only reported in 2 studies. Of the 12 nonrandomized trials, 10 did not report accounting for confounding variables in their design and analysis; however, representativeness in the study samples was generally adequate. The majority of the 12 quantitative descriptive studies’ methodologies were vulnerable to nonresponse bias.

#### Qualitative Studies

Studies’ methodologies were generally sufficient to meet the MMAT criteria for qualitative studies. It is worth noting, however, that most of the studies did not specifically outline the methodological framework underpinning the study, and it is unclear whether this was due to inadequate reporting or the absence of such structures entirely. Some studies did not appear to adequately substantiate their conclusions with the data, but again it was difficult to interpret whether this was due to flaws in methodology or omission of reporting.

### Synthesis of Results

#### Content of Digital Health Interventions for LGBTIQ+ Young People

Of the 5 interventions targeting mental health difficulties, 2 focused on internalizing symptom reduction [93-95], 1 targeted drug abuse prevention [92], and 2 focused on nonspecific aspects of psychological well-being [65,97]. Although few in number, existing digital mental health interventions targeted several LGBTIQ+ subgroups, and there was relatively little conceptual overlap between them. Four of these interventions were theory-driven, and only one mental health intervention showed noteworthy community involvement in the development of the intervention. With one exception, mental health interventions tended to rely on some form of skill-building or otherwise didactic content delivery.

There were few interventions targeting physical health problems or sexual health and well-being in LGBTIQ+ young people. Only one digital intervention focused on smoking cessation and targeted physical health in LGBTIQ+ young people [98,99]. This intervention showed aspects of both community-driven and theoretical designs. One digital intervention targeting sexual and reproductive well-being overall in LGBTIQ+ young people was developed with a theoretical framework but no community input [64]. Some aspects of this intervention overlapped with interventions targeting the risk of STIs, such as increasing condom use; however, other aspects diverged, such as including content on healthy relationships more broadly.

The majority of the interventions identified in this review were targeted toward risk reduction or the management of STIs. Of the 17 interventions identified as having focused on risk reduction and management of STIs, 1 focused on pre-exposure prophylaxis adherence [66], 2 focused on reducing unprotected sex [67-71], 7 targeted multiple aspects of HIV prevention [72-83], 2 focused on HPV prevention [84,85,101], 3 focused on increasing STI testing [86-89], and 2 focused on antiretroviral medication adherence [90,91]. All 17 interventions were targeted toward young men who are attracted to men, 2 of which were specifically for adolescents. The majority of these interventions included some aspect of community involvement in their design, although the extent varied from iterations based on user feedback to more central participatory design, which few interventions involved. All but one intervention was theory-driven in nature, with most interventions drawing from either the Information-Motivation-Behavioral Skills Model or Social Cognitive Theory in their design. Therefore, there was notable conceptual overlap among these interventions, which largely varied only in their delivery format and the breadth of their focus (ie, targeting specific aspects of risk reduction, such as condom use, versus a variety of such behaviors).

#### Delivery of Digital Health Interventions for LGBTIQ+ Young People

The most common platforms for digital health interventions were websites and mobile apps that, combined, represented over half of the interventions identified. A smaller number of interventions were delivered via computer software. Many interventions used gamification, or elements of game playing such as point scoring, in their delivery; however, only 2 interventions were fully gamified in nature [90,95]. Few interventions incorporated social interactivity and where present, they were typically minimal [72,75,78,90,98]. Most interventions were multimedia, incorporating a number of different delivery formats and types of content.

The vast majority of interventions delivered information to effect change either in terms of building awareness about the health issue in question or teaching skills to enable behavior change. This information is typically delivered via text or videos. For some interventions, this was the entirety of their scope; however, others included quizzes, games, or practice scenarios to consolidate the knowledge being presented. Interventions varied significantly in the extent of their personalization; some interventions delivered the same content to all participants, while others provided opportunities for personalized input and then delivered information specific to the individual’s situation or needs at the time.

Interventions varied in the duration and intensity of their delivery. In total, 16 of the 24 interventions appeared to be intended to be a perpetually available resource that could be accessed at any time and largely completed in a single instance if desired. Intervention duration ranged from very brief completion times, as low as 10 min [84], to up to 3.5 hours [95] to complete. In total, 8 of the 24 interventions staggered their delivery in some respect, such as presenting new content over a certain period [66,90], or otherwise incentivizing users to return to the intervention over a period of up to several months [96]. Few interventions described periods of use over 2 months; however, this may reflect the limitations of trial periods rather than their ideal dissemination. Overall, a small minority of interventions appear to be available for public use at present.

#### Effectiveness of Digital Health Interventions for LGBTIQ+ Young People

With regard to the effectiveness of these interventions, there was consistent evidence from a number of interventions that digital health interventions could improve HIV testing rates in young MSM [79,80,88,89]. Aside from this, STI-focused interventions appeared to effect change more consistently in cognitive or attitudinal outcomes, such as HIV awareness and preparedness to use condoms, than in behavioral outcomes in practice, such as condom use and unprotected anal sex. Some studies observed changes in these outcomes [69,73,74,83], while others did not [67,79,80]. There was insufficient evidence to suggest that interventions targeting a specific risk-related outcome were more effective at improving that outcome than interventions that sought change in a variety of risk-related outcomes.

Comparatively, few interventions were targeted toward improvement of mental or physical health issues; however, the interventions that did exist in this sphere were more targeted and overlapped less in scope. With the exception of an expressive writing intervention [97], digital health interventions demonstrated preliminary effectiveness in reducing internalizing symptoms such as depression and psychological distress [65,95]. Digital health interventions have also been reported to be effective at reducing substance use, including cigarette use [99] and peer drug use [92]. Owing to the limited number of interventions targeting these difficulties, it is difficult to determine any patterns regarding the factors predicting greater effectiveness; however, the only intervention that did not show notable effectiveness was also the only intervention to not present any didactic or skill-building content to the user.

Given that the majority of the interventions included in the review were multimedia in some form, it was not possible to draw conclusions about the delivery aspects that would most reliably effect change in outcomes. Furthermore, and importantly, given that the overall quality of the interventions included in this review was suboptimal, their effectiveness must be viewed in light of limitations associated with methodology and reporting.

#### Acceptability and Feasibility of Digital Health Interventions for LGBTIQ+ Young People

Overall, digital health interventions were generally acceptable to LGBTIQ+ young people, and there were some clear themes in aspects of these interventions that determined users’ level of interest. Gamification stood out as a component of interventions that tended to be highly regarded by participants. Information presented with brevity and in a relatable way tended to receive greater ratings of acceptability from users and, although infrequent, social aspects of interventions, such as the ability to share experiences with others, were generally highly rated as well. Common concerns raised about the interventions included information being too text heavy, patronizing or contrived, and tasks feeling too laborious or *homework-like*. Importantly, users voiced concerns regarding the targeting of the interventions to LGBTIQ+ people, for example, being targeted at a superficial level, or coming across as stereotypical and alienating in its presentation of LGBTIQ+ people.

Regarding the feasibility of the interventions, measures of engagement and adherence were often not reported by the included studies; however, those that did report levels of use (eg, screen time, clicks, communication with other users) appeared adequate, given the intended scope of the intervention. One study that included an in-person component (collecting rewards earned in the web-based component) reported low user engagement with this feature (5%-27%). Overall, rates of attrition among the included studies were low, with several interventions reporting retention rates of 90% to 100% in their trials [66,69,77]. Notably, however, a trial of one of these interventions in a community setting reported a much lower retention rate of 45.4% [67], indicating that engagement may be lower in reality than controlled trials would suggest. Other studies reported retention rates of 70% to 90%. Two other studies reported notably higher rates of attrition than the others [65,89]. These high rates of attrition did not appear linked to acceptability, with both interventions reporting largely positive responses from participants. There were no notable differences in acceptability and feasibility based on the health outcome interventions.

## Discussion

LGBTIQ+ young people have a substantially higher risk of a range of health difficulties than the general population [1], and targeted digital health interventions have the potential to play a crucial role in reducing these disparities [30,33]. The aims of this review were to (1) synthesize the scope of evidence-based, targeted digital health interventions that exist for this population, (2) to identify the overall effectiveness, acceptability, and feasibility of these interventions in this population, and (3) to provide recommendations for their development. The review identified many interventions designed to improve health in LGBTIQ+ young people, and these interventions have shown preliminary effectiveness in producing changes in some health outcomes in this group. Particularly promising evidence was found for the effectiveness of digital health interventions in certain aspects of managing the risk of STIs, notably increasing HIV testing rates, and emerging evidence was also found for reductions in internalizing symptoms and substance use. The review observed a trend that digital health interventions for LGBTIQ+ young people may more consistently effect change in cognitive and affective outcomes than behavioral outcomes, though this was not prescriptive. All of these findings must be considered in light of the preliminary nature of the majority of the studies included in this review and their resultant methodological limitations.

In addition to showing potential for effectiveness, the interventions were found to be generally acceptable and feasible overall. Acceptability appeared closely linked to collaborative intervention design development with LGBTIQ+ young people and the digital modality of delivery. Notably, one study, which had regular check-ins with a clinician, found that participants were in fact deterred by this contact, citing difficulty scheduling and desire to remain discreet [96]. There is currently insufficient evidence to conclude that digital health interventions would be more effective than untargeted or face-to-face interventions in controlled conditions, as most of the studies outlined in this review did not provide such comparisons. However, results such as these suggest that digital health interventions may not need to be more effective than other forms of intervention to be valuable; these interventions would likely engage sections of the LGBTIQ+ youth population who would otherwise be deterred from seeking any support at all. In addition, rates of attrition in the included interventions were lower than those reported in similar interventions for other groups [37,39-41]. When combined with the innately greater potential for dissemination and cost effectiveness that comes with the digital medium, these findings support the premise that digital health interventions may be an important avenue for reducing health disparities in LGBTIQ+ young people in the future.

### Future Directions

Overall, the results of this review are therefore promising for the continued development of digital health interventions for LGBTIQ+ young people, and there are some clear paths forward for how this field of research could be developed further. Most of the interventions included in this review have thus far only been evaluated in terms of usability, acceptability, and preliminary efficacy, and due to the preliminary nature of most of these studies, when efficacy was evaluated, aspects of methodology such as randomization and blinding were often lacking in rigor. The findings of this review are therefore consistent with others in the literature that frequently report low quality of evidence associated with digital health interventions [43,45,102,103]. In addition, only 1 study trialed a digital health intervention in a community setting [61], and its rate of user retention was approximately half that of its clinical trial, consistent with previous studies that have found a similar pattern [104]. The generalizability of the findings of these studies to the wider population of LGBTIQ+ young people, therefore, cannot be determined. There is a clear need to build on the emerging evidence base through more rigorous randomized controlled trials and trials in community settings, as this evidence base is crucial for further funding and dissemination. While the evidence thus far is promising, it needs significant development.

Outside of these methodological concerns, there is also notable progress in terms of expanding the scope of the digital health interventions that exist for this population. Most of the interventions identified in this review were directed toward improving the health of young men who identify as gay, bisexual, and queer and largely within the scope of improving sexual health–related concerns alone. Given the high rates of mental and physical health difficulties in LGBTIQ+ people, resources should be directed toward the development of digital health interventions targeting these issues, commensurate with the attention being given to STI and other sexual health–related concerns. The health concerns faced by LBTIQ+ women, trans and gender-diverse people, and people with intersex variations demand attention as well. Trans and gender-diverse people in particular face increased and wide-ranging health difficulties and barriers to health care access, even when compared with other members of the LGBTIQ+ community [25,27,105], and their marked underrepresentation in the interventions included in this review reveals a significant missed opportunity to begin to address these inequalities. While minority subpopulations are typically numerically small, and some argue that developing tailored interventions for such groups may be unnecessary [106], there is also evidence to suggest that minority groups appreciate tailoring of interventions. For example, in a recent study examining the attitudes of transgender and gender-diverse young people toward mental health gaming interventions, participants noted that TGD representation and inclusion of meaningful, specific tailored content was favorable [31].

### Limitations

This review is the first to provide a wide overview of digital health interventions for LGBTIQ+ young people, enabling gaps such as these to be highlighted. However, the review is limited in several ways. Owing to the restrictions placed on the degree of human guidance permissible for inclusion in the review, telehealth interventions are notably absent from those discussed here. The review also did not cover interventions for which their development has been documented but has not yet been evaluated in some respects, and the review also did not include studies documenting the effectiveness of nontargeted interventions for LGBTIQ+ young people. Furthermore, the requirement for included studies to be published in the English language may have resulted in a biased sample, potentially excluding reports on interventions published in other languages. Finally, it is possible that limiting the search to studies published after January 1, 2000, may have excluded relevant studies; however, given that the earliest published study identified in the review was not until 10 years later, this is unlikely to have been the case. The review therefore largely covers the scope of interventions that accumulate an evidence base but should not be taken to cover any and all digital health interventions that may benefit the health of LGBTIQ+ youth.

### Conclusions

Although not sought out specifically in the process of conducting this review, many protocols have been identified for continued research into the development of digital health interventions [107-112]. The development of digital health interventions for LGBTIQ+ young people is a burgeoning field of study, and we expect the evidence base to advance quickly. Going forward, this advancement should ideally occur across the breadth of health difficulties and inequalities that the entire LGBTIQ+ community faces and with appropriate methodological rigor. Given the number of interventions already targeting risk reduction or management of STIs, rather than developing more, future studies should ideally seek to refine and adapt those that exist for public use, and explore implementation barriers and facilitators to enhance translation. Given the lower scope and evidence base for interventions targeting mental and physical health difficulties, future studies should focus predominantly on expanding the available interventions and evidence base in these domains, particularly in terms of addressing difficulties such as alcohol use and suicide for which no digital health interventions were detected in this review at all. Digital health interventions for LGBTIQ+ young people show the potential to improve health disparities in this population, and the expansion of research along these lines is crucial to realize this potential.

## Appendix

Multimedia Appendix 1 Search strategy for PsycINFO (Ovid).

Multimedia Appendix 2 Summary of digital sexually transmitted infection risk reduction and management interventions for LGBTIQ+ young people.

Multimedia Appendix 3 Summary of interventions.

## Abbreviations

HPV: human papillomavirus
LGBTIQ+: lesbian, gay, bisexual, transgender, intersex or queer
MMAT: mixed methods appraisal tool
MSM: men who have sex with men
PrEP: pre-exposure prophylaxis
PRISMA: Preferred Reporting Items for Systematic Reviews and Meta-Analyses
PROSPERO: Prospective Register of Systematic Reviews
STI: sexually transmitted infection
