# Supplementary material for: Effectiveness, Acceptability, and Feasibility of Digital Health Interventions for LGBTIQ+ Young People: Systematic Review
Source: J Med Internet Res. 2020 Dec 3;22(12):e20158. doi: 10.2196/20158 (PMC7746499; doi:10.2196/20158)
Supplement: Multimedia Appendix 1 [file jmir_v22i12e20158_app1.docx]

**Multimedia Appendix 1**

The search of PsycINFO (Ovid) was conducted using a combination of (exploded) subject headings (Gender Identity, Sexual Orientation, Childhood Development, Adolescent Development, College Students, Junior High School Students, High School Students, Middle School Students, Mental Health, Well Being, Physical Health, Health Promotion, Health, Sexually Transmitted Diseases, Sexual Health, HIV, Sex Education, Electronic Health Services, Treatment or Prevention) and the keywords listed in the table below. The keyword searches were restricted to titles and/or abstracts.

| androphil* or androsexual* or bicurious or bisexual* or cross#sex or crossgender or disorder* in sex* development or difference* in sex* development or DSD* or F2M or gay* or gender change or gender dysphoria or gender identity or gender queer or gender reassign* or gender transform* or gender transition* or genderqueer or GLB* or gynephile* or gynesexual* or hermaphrodit* or heteroflexible or homosexual* or intersex* or lesbian* or lesbigay or LGB* or M2F or men who have sex with men or MSM or pansexual* or queer or same#gender loving or same#sex attracted or same#sex couple* or same#sex relations or sex change* or sex reassign* or sex reversal or sex transform* or sex transition* or (sexual and gender minorit*) or sexual identity or sexual minorit* or sexual orientation or sexual preference or SGM or trans female or trans male or trans m#n or trans people or trans person or trans wom#n or trans-sexuality or transexual or transgender* or transsexual* or women loving women or women who have sex with women or WSW or YMSM or YWSW |
| --- |
| adolescen* or child* or student* or minor or teen* or youth or young |
| anorex* or anxiety or anxious or binge* or bipolar or borderline personality or BPD or bulimi* or clinical or coping or depress* or cyclothymi* or disorder or distress or dysthym* or externali#ing or GAD or internali#ing or mania or mental health or mental illness or mental wellbeing or MDD or mindful* or mood or obsessive-compulsive or OCD or panic or phobi* or post-traumatic or psych* or PTSD or resilien* or self#compassion or self#harm or self#injur* or schiz* or stress or suicid* or trauma* |
| alcohol* or ATOD or blood pressure or BMI or body mass index or cancer or cardiovascular or cholesterol or chronic illness or cigarette or cocaine or CVD or dental or diabet* or diet* or disease* or drug or exercise or fat or hazardous drinking or hepatitis or hypertension or injur* or medication adherence or meth* or nutrition or obes* or physical activity or physical health or smok* or substance or tobacco or weight-reduction or vaccin* |
| acquired immunodeficiency syndrome or AIDS or antiretroviral or bareback* or BBV or blood borne virus* or chemsex or condom* or contracept* or (healthy adj3 relationship*) or HIV* or HPV or human papillomavirus or human immunodeficiency virus or PEP or post-exposure prophylaxis or PrEP or pre-exposure prophylaxis or reproductive health or safe sex or sex* education or sexual behaviour or sexual health or sexual* transmi* or STD or STI or teen* pregnancy or Truvada or unplanned pregnancy or unprotected sex |
| app or blog or cell phone or cellphone or computer* or cyber* or DBCI or digital health or digital health intervention or eHealth or electronic or email or e-therapy or Facebook or forum or gamification or gaming or Instagram or internet or mHealth or M-Health or mobile or online or serious game or smartphone or SMS or social media or social network or technol* or telemedicine or Tumblr or Twitter or web* or www or video gam* or virtual or VR |
| acceptability or acceptable or effectiveness or efficac* or feasib* or interven* or pilot or prevent* or program or randomi#ed controlled or RCT or therapeutic or therapy or treat* or trial or usability |
