# Supplementary material for: Effectiveness, Acceptability, and Feasibility of Digital Health Interventions for LGBTIQ+ Young People: Systematic Review
Source: J Med Internet Res. 2020 Dec 3;22(12):e20158. doi: 10.2196/20158 (PMC7746499; doi:10.2196/20158)
Supplement: Multimedia Appendix 2 [file jmir_v22i12e20158_app2.docx]

**Multimedia Appendix 2**

| \| Intervention name \| Primary health outcome \| Study \| Participant mean age (range) (intervention condition) \| Description of sample (e.g. LGBTIQ status/identification) \| Sample size \| Study design \| Study setting \| Intervention type \| Digital platform \| Primary measured constructs and effects \| \| --- \| --- \| --- \| --- \| --- \| --- \| --- \| --- \| --- \| --- \| --- \|   Pre-exposure prophylaxis (PrEP) adherence | | | | | | | | | | |
| --- | --- | --- | --- | --- | --- | --- | --- | --- | --- | --- | --- | --- | --- | --- | --- | --- | --- | --- | --- | --- | --- |
| mSMART [66] | Pre-exposure prophylaxis adherence | Mitchell et al. (2018) | 24.1 (18-30) | Young men who have sex with men. | 10 | Mixed-methods, one-arm pilot, feasibility, acceptability and efficacy | United States of America | Mobile app | Mobile phone | N/A |
| Reducing unprotected sex | | | | | | | | | | |
| Keep It Up! [67-70] | Reducing unprotected sex | Mustanski et al. (2013) | 21.6 (Range not provided) | Young men who have sex with men. 84=Gay, 18=Bisexual/other | 102 (50 Intervention, 52 control) | Mixed-methods, pilot RCT and follow-up, efficacy and acceptability | United States of America | Multimedia website | Computer or tablet via web | Unprotected anal sex ↓ |
|  |  | Greene et al. (2016) | 21.5 (Range not provided) | Young men who have sex with men and trans women. Gay= 251, Bisexual/other= 90, Trans= 17 | 343 | Mixed-methods, one-arm trial and follow-up, effectiveness. Community-based service implementation. | United States of America - Community setting |  |  | Unprotected anal sex –  Unprotected anal sex with casual partners ↓  HIV Knowledge ↑  Condom errors ↓ |
|  |  | Mustanski et al. (2018) | Mean not provided (18-29) | Young men who have sex with men. | 901 (445 Intervention, 456 Control) | Quantitative, RCT and follow-up, efficacy | United States of America |  |  | STI incidence ↓  Unprotected anal sex ↓ |
|  |  | Madkins et al. (2019) | 24.3 (Range not provided) | Young men who have sex with men. 385=Gay, 60=Bisexual | 901 (445 Intervention, 456 Control) | Mixed-methods, RCT, acceptability data |  |  |  | N/A |
| SOLVE [71] | Reducing unprotected sex | Christensen et al. (2013) | 21.3 (18-24) | Young men who have sex with men. Gay=707, Bisexual=111, Other=103 | 921 (491 Intervention, 444 Control) | Quantitative, RCT and follow-up, efficacy | United States of America | Serious game | Computer | Direct change in unprotected anal sex not reported. |
| HIV prevention: multiple outcomes | | | | | | | | | | |
| HealthMpowerment [72-74] | HIV risk reduction | Hightow-Weidman et al. (2011) | 22.4 (18-30) | Young black men who have sex with men. | 7 | Qualitative, usability testing | United States of America | Multimedia website | Computer via web | N/A |
|  |  | Hightow-Weidman et al. (2012) | 25.0 (19-30) | Young black men who have sex with men. 31= Gay, 15= Bisexual, 2= Other | 50 (25 Intervention, 25 Control) | Mixed-methods, controlled pilot, efficacy and acceptability testing |  |  |  | Condom use ↑  HIV knowledge – |
|  |  | Hightow-Weidman et al. (2019) | 24.3 (18-30) | Young black men who have sex with men. 316= Gay, 95= Bisexual, 63=Other | 474 | Quantitative, efficacy, RCT and follow-up |  |  |  | Unprotected anal sex ↓ |
| Unnamed intervention [75] | HIV prevention | Ybarra et al. (2014) | 16.4 (14-18) | Teenage gay, bisexual and queer men. Gay=65, Bisexual=14, Queer=4 | 75 | Qualitative, focus groups, feasibility | United States of America | Online focus group discussions | Mobile phone | N/A |
| Guy2Guy [76-79] | HIV prevention | Ybarra et al. (2016) | Mean not provided (14-18) | Adolescent gay, bisexual and queer men. Gay=49, Bisexual=5, Queer=6 (some participants identified with more than one category) | 52 (32 in content advisory teams, 18 in beta test) | Mixed-methods, acceptability and feasibility testing | United States of America | Text messaging | Mobile phone | N/A |
|  |  | Ybarra et al. (2017) | Mean not provided (14-18) | Adolescent gay, bisexual and queer men. Gay=203, Other= 80 | 283 (137 Intervention, 146 Control) | Quantitative, pilot RCT, efficacy |  |  |  | Unprotected anal sex –  HIV testing ↑ |
|  |  | Ybarra et al. (2018) | 16.0 (14-18) | Adolescent gay, bisexual and queer men. | 273 (133 Intervention, 140 Control) | Quantitative, RCT, efficacy |  |  |  | Condom use skills –  HIV knowledge ↑ |
|  |  | Ybarra et al. (2019) | 16.0 (14-18) | Adolescent gay, bisexual and queer men. | 275 (132 Intervention, 133 Control) | Mixed-methods, RCT, survey, focus groups, acceptability and feasibility |  |  |  | N/A |
| Tu Amigo Pepe [80] | HIV prevention | Solorio et al. (2016) | 25.0 (18-30) | Young, Latino men who have sex with men. Gay=34, Bisexual/other=10, Straight=5 | 50 | Quantitative, one-arm pilot, efficacy | United States of America | Multimedia website | Computer via web | HIV testing ↑  Condom use – |
| MyPEEPS Mobile [81] | HIV prevention | Cho et al. (2018) | 17.4 (15-18) | Teenage, racially and ethnically diverse men who have sex with men. Gay=14, Mostly gay=2, Bisexual=3, Something Else=1 | 20 | Mixed-methods, usability testing | United States of America | Mobile app | Mobile phone | N/A |
| Tough Talks [82] | HIV risk reduction | Muessig et al. (2018) | 25.0 (20-28) | Young men who have sex with men. Gay= 6, Bisexual= 5 | 11 | Quantitative, one-arm pilot, acceptability and efficacy | United States of America | Virtual reality | Computer | N/A |
| myDEx [83] | HIV prevention | Bauermeister et al. (2019) | 21.5 (18-24) | Young gay and bisexual men who have sex with men. Gay= 138, Bisexual= 11, Queer= 6 | 155 | Quantitative, pilot RCT, acceptability and efficacy | United States of America | Multimedia mobile app | Mobile phone | Unprotected receptive anal sex ↓ |
| HPV prevention | | | | | | | | | | |
| Unnamed intervention [84] | HPV vaccination and screening | Burnham (2017) | 23.5 (18-26) | Young men who have sex with men. Men= 99, Trans women=4 | 103 | Mixed-methods, one-arm pilot, acceptability and efficacy | United States of America | e-Health video | Web | N/A |
| Outsmart HPV [85, 101] | HPV vaccination | McRee et al. (2018) | Mean not provided (18-25) | Young men who have sex with men. Gay=118, Bisexual=23 | 141 (68 Intervention, 73 Control) | Quantitative, pilot RCT, acceptability and efficacy | United States of America | Website | Computer, tablet or mobile phone | HPV testing ↑ (n.s.) |
|  |  | Reiter et al. (2018) | Mean not provided (18-25) | Young men who have sex with men. Gay=124, Bisexual=26 | 150 (76 Intervention, 72 Control) | Quantitative, pilot RCT and follow-up, efficacy |  |  |  | HPV vaccine initiation ↑  HPV vaccine completion - |
| *STI testing* | | | | | | | | | | |
| Get Connected! [86, 87] | STI testing | Bauermeister et al. (2015) | 21.1 (15-24) | Young men who have sex with men. Gay=109, Bisexual=19, Other=2 | 130 (86 Intervention, 44 Control) | Quantitative, pilot RCT, efficacy | United States of America | Website | Computer via web | STI testing ↑ (n.s.) |
|  |  | Horvath & Bauermeister (2017) | 21.1 (15-24) | Young men who have sex with men. Gay=109, Bisexual=19, Other=2 | 130 (86 Intervention, 44 Control) | Quantitative, pilot RCT, acceptability |  |  |  | N/A |
| Unnamed intervention [88] | HIV testing | Washington et al. (2017) | 23 (18-30) | Young black men who have sex with men. | 42 | Quantitative, pilot RCT and follow-up, feasibility | United States of America | Videos presented via social media (Facebook) | Web | HIV testing ↑ |
| Stick To It [89] | HIV testing | McCoy et al. (2018) | 23.0 (18-26) | Young men who have sex with men. Only male sex partners=138, Male & female sex partners=28 | 166 | Mixed-methods, one-arm pilot and follow-up, acceptability and efficacy | United States of America | Gamified website | Web | HIV testing ↑ |
| Antiretroviral medication adherence | | | | | | | | | | |
| Epic Allies [90] | Antiretroviral therapy adherence | LeGrand et al. (2016) | 23.0 (20-28) | HIV-positive young men who have sex with men. | 7 | Mixed-methods, usability testing | United States of America | App/ Serious Game | Mobile phone | N/A |
| AllyQuest [91] | Antiretroviral therapy adherence | Hightow-Weidman et al. (2018) | 21.8 (19-24) | Young, HIV-positive men who have sex with men. Gay= 19, Bisexual= 1 | 20 | Mixed-methods, one-arm pilot, usability and efficacy | United States of America | Mobile app | Mobile phone | N/A |

– No change

↑ Significant increase

↓ Significant decrease

66. Mitchell, J.T., et al., *Smartphone-Based Contingency Management Intervention to Improve Pre-Exposure Prophylaxis Adherence: Pilot Trial.* JMIR Mhealth Uhealth, 2018. **6**(9): p. e10456.

67. Greene, G.J., et al., *Implementation and Evaluation of the Keep It Up! Online HIV Prevention Intervention in a Community-Based Setting.* AIDS Education & Prevention, 2016. **28**(3): p. 231-45.

68. Madkins, K., et al., *Measuring Acceptability and Engagement of The Keep It Up! Internet-Based HIV Prevention Randomized Controlled Trial for Young Men Who Have Sex With Men.* AIDS Education & Prevention, 2019. **31**(4): p. 287-305.

69. Mustanski, B., et al., *Feasibility, acceptability, and preliminary efficacy of an online HIV prevention program for diverse young men who have sex with men: the keep it up! intervention.* AIDS and Behavior, 2013. **17**(9): p. 2999-3012.

70. Mustanski, B., et al., *Biomedical and Behavioral Outcomes of Keep It Up!: An eHealth HIV Prevention Program RCT.* American journal of preventive medicine, 2018.

71. Christensen, J.L., et al., *Reducing shame in a game that predicts HIV risk reduction for young adult MSM: a randomized trial delivered nationally over the Web.* Journal of the International AIDS Society, 2013. **16**(3 Suppl 2): p. 18716.

72. Hightow-Weidman, L., et al., *HealthMpowerment.org: development of a theory-based HIV/STI website for young black MSM.* AIDS Education & Prevention, 2011. **23**(1): p. 1-12.

73. Hightow-Weidman, L., et al., *A Randomized Trial of an Online Risk Reduction Intervention for Young Black MSM.* AIDS & Behavior, 2019. **23**(5): p. 1166-1177.

74. Hightow-Weidman, L., et al., *HealthMpowerment. org: feasibility and acceptability of delivering an internet intervention to young Black men who have sex with men.* AIDS care, 2012. **24**(7): p. 910-920.

75. Ybarra, M.L., et al., *Online focus groups as an HIV prevention program for gay, bisexual, and queer adolescent males.* AIDS Education and Prevention, 2014. **26**(6): p. 554-564.

76. Ybarra, M.L., et al., *The Effect of a Text Messaging Based HIV Prevention Program on Sexual Minority Male Youths: A National Evaluation of Information, Motivation and Behavioral Skills in a Randomized Controlled Trial of Guy2Guy.* AIDS and behavior, 2018: p. 1-10.

77. Ybarra, M.L., et al., *Feasibility, Acceptability, and Process Indicators for Guy2Guy, an mHealth HIV Prevention Program for Sexual Minority Adolescent Boys.* Journal of Adolescent Health, 2019.

78. Ybarra, M.L., et al., *Iteratively developing an mHealth HIV prevention program for sexual minority adolescent men.* AIDS and Behavior, 2016. **20**(6): p. 1157-1172.

79. Ybarra, M.L., et al., *Pilot RCT results of an mHealth HIV prevention program for sexual minority male adolescents.* Pediatrics, 2017. **140**(1): p. e20162999.

80. Solorio, R., et al., *Tu Amigo Pepe: Evaluation of a Multi-media Marketing Campaign that Targets Young Latino Immigrant MSM with HIV Testing Messages.* AIDS & Behavior, 2016. **20**(9): p. 1973-88.

81. Cho, H., et al., *A Mobile Health Intervention for HIV Prevention Among Racially and Ethnically Diverse Young Men: Usability Evaluation.* JMIR mHealth and uHealth, 2018. **6**(9): p. e11450.

82. Muessig, K.E., et al., *"I Didn't Tell You Sooner Because I Didn't Know How to Handle It Myself." Developing a Virtual Reality Program to Support Hiv-Status Disclosure Decisions.* Digital Culture & Education, 2018. **10**: p. 22-48.

83. Bauermeister, J.A., et al., *Acceptability and Preliminary Efficacy of an Online HIV Prevention Intervention for Single Young Men Who Have Sex with Men Seeking Partners Online: The myDEx Project.* AIDS & Behavior, 2019. **14**: p. 14.

84. Burnham, B.M., *Addressing the Epidemic of Anal Human Papillomavirus (HPV) Infection via an E-Health Video Designed to Empower Young Men Who Have Sex With Men (YMSM) to Increase HPV Screening and Vaccination, Screening for HIV and Other Sexually Transmitted Infections, and Adoption of Risk Reduction Behaviors*. 2017, Teachers College, Columbia University: Ann Arbor. p. 259.

85. McRee, A.L., et al., *Outsmart HPV: Acceptability and short-term effects of a web-based HPV vaccination intervention for young adult gay and bisexual men.* Vaccine, 2018. **36**(52): p. 8158-8164.

86. Bauermeister, J.A., et al., *Acceptability and preliminary efficacy of a tailored online HIV/STI testing intervention for young men who have sex with men: the Get Connected! program.* AIDS and Behavior, 2015. **19**(10): p. 1860-1874.

87. Horvath, K.J. and J.A. Bauermeister, *eHealth literacy and intervention tailoring impacts the acceptability of a HIV/STI testing intervention and sexual decision making among young gay and bisexual men.* AIDS Education and Prevention, 2017. **29**(1): p. 14-23.

88. Washington, T.A., S. Applewhite, and W. Glenn, *Using Facebook as a Platform to Direct Young Black Men Who Have Sex With Men to a Video-Based HIV Testing Intervention: A Feasibility Study.* Urban Social Work, 2017. **1**(1): p. 36-52.

89. McCoy, S.I., et al., *Stick To It: pilot study results of an intervention using gamification to increase HIV screening among young men who have sex with men in California.* Mhealth, 2018. **4**.

90. LeGrand, S., et al., *Epic Allies: development of a gaming app to improve antiretroviral therapy adherence among young HIV-positive men who have sex with men.* JMIR serious games, 2016. **4**(1): p. e6.

91. Hightow-Weidman, L., et al., *A Gamified Smartphone App to Support Engagement in Care and Medication Adherence for HIV-Positive Young Men Who Have Sex With Men (AllyQuest): Development and Pilot Study.* JMIR Public Health and Surveillance, 2018. **4**(2): p. e34.

101. Reiter ,P.L. et al., *Increasing human papillomavirus vaccination among young gay and bisexual men: a randomized pilot trial of the outsmart HPV intervention*. LGBT Health, 2018. p. 325-329
