# Supplementary material for: Effectiveness, Acceptability, and Feasibility of Digital Health Interventions for LGBTIQ+ Young People: Systematic Review
Source: J Med Internet Res. 2020 Dec 3;22(12):e20158. doi: 10.2196/20158 (PMC7746499; doi:10.2196/20158)
Supplement: Multimedia Appendix 3 [file jmir_v22i12e20158_app3.docx]

**Multimedia Appendix 3**

***Evidence-based interventions for improving mental wellbeing and treatment of mental health concerns***

*Drug abuse prevention*

Unnamed intervention (Schwinn et al. 2015)

Schwinn et al. developed a brief, online multimedia intervention designed to reduce drug abuse in sexual minority youth [92]. The intervention involves interactive games, role-playing and writing activities to build skills related to drug use-related decision making. An RCT of the intervention found that users who participated reported less peer drug use (*d*=0.31) and other drug use (*d*=0.34) at 3-month follow-up relative to a control group receiving no intervention exposure; however, use of alcohol, cigarettes and marijuana did not differ by arm. Eight two per cent of participants completed all three components of the intervention over the required four weeks, indicating adequate feasibility. The acceptability of the intervention was not evaluated.

*Internalising disorder prevention/management*

Rainbow SPARX

Rainbow SPARX is an LGBT+ tailored adaptation of SPARX, a didactic serious game on PC designed to prevent depression in young people using cognitive behavioural therapy principles, such as behavioural activation, cognitive restructuring, and problem solving [100]. Rainbow SPARX was adapted based on focus group input on how SPARX may be improved for sexual minority youth [53, 93-95]. Although uptake was low, a pilot feasibility trial showed that participation in the game was associated with large decreases in depressive (*d*=1.01) and anxiety (*d*=0.95) symptoms and 81% of participants completed all modules. The intervention was reported to be generally acceptable to participants, however, some participants expressed concerns that the level of LGBT+ representation was too superficial, and the language and content felt patronising. Participants liked that the intervention was made especially for young people.

TODAY!

TODAY! is a didactic mobile app designed to reduce symptoms of depression and anxiety in young sexual minority men using transdiagnostic CBT concepts and skills such as cognitive restructuring and problem solving, in addition to brief, weekly support from a clinician [96]. Usability testing of the app has found participants to have mostly positive reactions to the app after ten weeks of use, however, participants endorsed the importance of the didactic components of the app being briefer and more concise. Participants also requested more content specifically tailored to gay and bisexual men. One of the biggest barriers to usability was the required contact with a clinician, with participants citing concerns about scheduling and desire to be discreet.

*Non-specific mental health interventions*

Unnamed intervention (Pachankis & Goldfried, 2010)

Pachankis and Goldfried (2010) tested the efficacy of an online expressive writing intervention to improve psychosocial functioning in gay male college students, asking participants to write about the most stressful or traumatic event related to their sexuality they had experienced over three twenty-minute daily writing sessions [97]. In an RCT, no differences in depressive symptoms or other symptoms of psychopathology were observed between the intervention group and control post-test and at 3-month follow-up.

QueerViBE

QueerViBE is a series of brief (6-12 minute), online, interactive video tutorials designed to improve wellbeing in trans male and non-binary youth by validating trans and non-binary identities and guiding responses to intrusive questions and misgendering [65]. An RCT of the intervention found that participation in QueerViBE resulted in a moderate decrease in psychological distress (*d*=0.63). Participants in the intervention reported that they found the videos interesting, relevant and easy to understand; however, attrition from the intervention was very high (77.5%).

***Evidence-based interventions for improving physical wellbeing and treatment of physical health concerns***

POP (Put it Out Project)

POP is a social media-based smoking cessation intervention for sexual and gender minority young adults [98, 99]. The intervention consists of private Facebook groups wherein content is posted that includes motivational interviewing, experiential and behavioural strategies to assist with smoking cessation. The content of these posts was informed by qualitative data on user preferences. Users can also interact with a smoking cessation counsellor in the group once a week. A pilot RCT of the intervention found that involvement in POP reduced self-reported weekly cigarette use and 7-day abstinence relative to an untailored version of the intervention after three months. The intervention was found to be viewed positively by participants overall, however, no difference in acceptability was observed between POP and the untailored control version. Eight one per cent of users commented on the posts at least once, and the mean number of comments per user was 52, indicating that the posts were able to engage users.

***Evidence-based interventions for improving sexual and reproductive wellbeing***

Queer Sex Ed

Queer Sex Ed is an online didactic intervention designed to promote sexual health and wellbeing in LGBT youth [64]. The intervention includes five modules teaching participants about topics such as forming and sustaining healthy relationships, safe sex and developing personalised sexual health improvement goals. An initial trial of the intervention found that participation resulted in moderate increases in most sexual health variables, including sexual functioning (*d*=0.27), contraceptives knowledge (*d*=0.39) and STI knowledge (*d*=0.34), and small increases in sexual assertiveness (*d*=0.11) and communication skills (*d*=0.08). Participant attitudes towards the intervention were largely positive, particularly regarding the inclusion of information about relationships and sexual functioning in lieu of focusing solely on STIs. Concerns were raised about insufficient tailoring of the program content, for example, having limited trans-relevant information.

***Evidence-based interventions for risk reduction and management of sexually transmitted infections***

*Pre-exposure prophylaxis (PrEP) adherence.*

mSMART

mSMART is a smartphone-based contingency management intervention designed to improve adherence to PrEP in young men who have sex with men [66]. The primary functions of the app are medication reminders and daily logging of medication use, with contingent reinforcement of $2 USD when doses are logged. Experts, but not users, were consulted during the development of mSMART. A pilot trial of the intervention found that PrEP adherence increased for only 30% of the sample; however, 90% of the sample already had acceptable medication adherence at baseline and ceiling effects were therefore likely present. Usability of the intervention was rated as moderate and user satisfaction was less than moderate, with users noting that the app was too text heavy. Despite this, there was no user attrition and daily engagement with the logging feature of the app was high (91%), indicating feasibility.

*Reducing unprotected sex.*

Keep it Up!

Keep it Up! is an online HIV prevention program designed for young men who have sex with men [67-70]. The intervention uses a variety of media, including peer videos, animated games, and plan development to address gaps in HIV knowledge and teach preventive behaviours. In an RCT, Keep it Up! was found to be associated with a 68% relative reduction in STI incidence compared to an online HIV knowledge control condition 12-months post-intervention. Another trial of the intervention in a community-based setting observed moderate decreases in casual sexual partners (*d*=0.37) and condomless anal sexual intercourse with casual sexual partners (*d*= 0.20), among other effects. Participants described liking the intervention content overall and its tailoring was particularly well-received. Completion rates were high in controlled trials of the intervention (84-96%), but lower in a community-based setting (45%).

Socially Optimised Learning in Virtual Environments (SOLVE)

SOLVE is a simulation-based serious game designed to reduce HIV risk by reducing shame in young adult men who have sex with men [71]. The game involves immersion in a virtual world where players are confronted with decisions related to sexual health risk, after which they are exposed to didactic messages related to skills contextualised to appropriately navigating the scenario in question. Several population-matched community advisory boards informed the design process. An RCT found that the intervention had no direct effect on reducing unprotected anal intercourse, however, the intervention led to reductions in shame (*d*=0.29) which indirectly predicted reduction in unprotected anal intercourse.

*HIV prevention: multiple outcomes.*

HealthMpowerment.

HealthMpowerment is a multimedia, online intervention designed to reduce risk of HIV/STI infection in young, black men who have sex with men [72-74]. The intervention is a website consisting of health information, peer videos and stories, strategies to address barriers to behaviour change, quizzes and a forum, among other components. The intervention was designed based on focus group data and has been refined based on two rounds of usability testing. An RCT of the intervention found evidence that it is effective in reducing rates of condomless anal intercourse. Rates of condomless anal intercourse were 32% lower among the intervention group at 3-month follow-up compared to a control website; however, this difference was not maintained to 12-month follow-up [73]. A previous trial observed no group differences in a variety of HIV risk-related factors post-intervention [74]. User satisfaction with the HealthMpowerment site was high, as were rates of user retention (78-85%) [80, 82].

Unnamed intervention (Ybarra et al., 2014)

Ybarra, DuBois [75] explored the potential of online focus group discussions of HIV prevention programs as a medium for HIV prevention among gay, bisexual and queer adolescent males. Participants in the focus groups reported positive changes in their views on sex and condom use and likelihood of positive behaviour changes; sexually inexperienced participants felt that their participation in the focus groups was beneficial to them. The efficacy of focus groups as an HIV prevention intervention has not been explored further.

Guy2Guy

Guy2Guy is a gamified text messaging-based HIV prevention program for sexual minority adolescents [76-79]. Users receive 8-15 messages per day containing HIV-prevention related information, motivation and behavioural skills, and quizzes on this information, as well as being paired with another user to converse with, among other features. Guy2Guy was developed with heavy user involvement in the form of focus groups, content advisory teams and beta testers. In an RCT, evidence was found that Guy2Guy was effective at increasing motivation to engage in HIV preventive actions, but not at enhancing HIV knowledge or use of behavioural skills for abstinence or condom use, relative to a non-gamified control. A pilot RCT previously documented evidence that the intervention may increase likelihood of getting tested for HIV. The intervention was found to be highly acceptable to participants, and the rate of user retention was high (94%).

Tu Amigo Pepe

Tu Amigo Pepe is a multimedia campaign designed to increase HIV testing and condom use in young, Latino men who have sex with men [80]. A major component of the campaign is a website designed to increase intentions for HIV-preventive actions predominantly by influencing attitudes, norms and self-efficacy surrounding these behaviours. The website provides HIV information, an HIV test locator, and peer videos among other features. Other components of Tu Amigo Pepe, such as radio announcements, were developed with participatory action, but it is unclear whether users were involved in the development of the website. A pilot trial of the intervention found evidence that it may be effective at increasing HIV testing rates and attitudes towards condom use, but not condom use rates in practice.

MyPEEPS Mobile

MyPEEPS is a didactic mobile app designed to prevent HIV in racially and ethnically diverse young men who have sex with men [81]. The app delivers HIV prevention information guided by four avatars, including videos, didactic content, quizzes and rewards for completion of activities. The mobile version of MyPEEPS was adapted using a participatory approach, involving and incorporating feedback from users and an expert panel. The efficacy of this intervention has not yet been evaluated; however, usability testing found the app to have strong user acceptance, citing positive attitudes to the design, layout, videos and quizzes.

Tough Talks

Tough Talks is a virtual reality intervention designed to reduce HIV risk by assisting young gay and bisexual men with HIV to gain the self-efficacy to disclose their HIV serostatus to others [82]. Users practice role-playing disclosure with a variety of characters in different settings. Tough Talks was designed iteratively alongside user focus groups. The intervention has been found to be perceived positively by participants.

myDEx

myDEx is a web-based, multimedia HIV prevention intervention designed for young men who have sex with men, predominantly those seeking partners online [83]. The content of the intervention, presented across six sessions, includes videos, roleplay scenarios, quizzes and a diary to log experiences dating, together aiming to building HIV-risk reduction and enhance self-reflection. A youth advisory board was involved in the design process. In a pilot trial, young men who participated in myDEx were found to be less likely than control participants to have engaged in condomless receptive anal sex during the 3-month trial period (*d*=0.48); however, there were no group differences in HIV testing, number of sexual partners or safe-sex self-efficacy. Participants in myDEx were more likely than untailored control participants to report that the intervention was useful and acceptable.

*HPV prevention.*

Unnamed intervention (Burnham, 2017)

Burnham [84] developed a brief, 10-minute eHealth video designed to increase HPV screening and vaccination among young men who have sex with men. The video presents information related to screening, vaccination, refusing unprotected sex and negotiating condom use in an informal narrative format. In a pilot trial, watching the video was found to lead to higher motivation to change current levels of risk reduction behaviours, in addition to significantly higher self-efficacy to perform these behaviours. It has not yet been trialled whether the intervention practically increases HPV screening rates. The video was found to be acceptable to participants overall, citing that it was informative, accessible and relatable; however, there were concerns that the acting was “cheesy” and forced and that the content was not engaging.

Outsmart HPV

Outsmart HPV is a web-based intervention designed to improve rates of HPV vaccination in young gay and bisexual men [85, 101]. The website, designed with user input, presents tailored information relating to HPV and its vaccine, culminating in the development of a personalised vaccination plan. In a pilot RCT, Outsmart HPV was found to be associated with greater likelihood of initiating the HPV vaccine and of speaking to a healthcare provider about the vaccine. A prior pilot study also found evidence that the intervention led to lower perceived harms of the vaccine (*d*=0.23) and increased perceived risk of anal cancer (*d*=0.30), in addition to the intervention being highly acceptable to participants.

*STI testing*

Get Connected!

Get Connected! is a website designed to improve HIV/STI testing rates in young men who have sex with men predominantly by directing them to testing sites based on input user information (e.g. race/ethnicity, age, relationship status) [86, 87]. The website assesses user motivations, attitudes, values and strengths regarding testing, and explores barriers before providing personalised testing recommendations based on this assessment. The thematic design of Get Connected! was heavily influenced by input from a youth advisory board. A pilot RCT found evidence that individuals who used Get Connected! were more likely than control participants to have made an appointment for testing at 3-month follow-up to a clinical meaningful (*d*=0.34), but not statistically significant degree. The intervention was found to be highly acceptable, and the tailored information presented by Get Connected! was perceived as more accurate than the non-tailored control.

Unnamed intervention (Washington et al., 2017)

Washington, Applewhite [88] tested the effectiveness of brief eHealth videos presented on Facebook at increasing HIV testing in young black men who have sex with men. The videos, delivered by peer actors, covered HIV prevention knowledge, the benefits of early detection of HIV and coping with disclosure of HIV, among other topics. The videos were refined based on user feedback. In a pilot RCT, they observed individuals who were assigned to watch the videos to have seven-times greater odds of having tested for HIV at 6-month follow-up, compared to a control group who received standard, untailored text information about HIV.

Stick to It

Stick to It is a gamified website designed to increase HIV screening among young men who have sex with men [89]. Website users complete online activities, such as quizzes, to earn points which can be redeemed for prizes in-person in an HIV testing clinic. Stick to It was designed through an iterative process involving user focus groups and clinic staff feedback. A pilot study of the intervention found evidence that use of Stick to It may improve rates of HIV testing; 48% of intervention participants received two or more HIV tests at the clinic over 6 months, compared to a rate of 30% in a historical comparison of young men who have sex with men at the clinic. Despite positive feedback and acceptability for the intervention, engagement was relatively low, with only 19% of participants completing an activity and significant drop-off after registration.

*Antiretroviral medication adherence.*

Epic Allies

Epic Allies is a serious game mobile app designed to increase antiretroviral medication adherence in young, HIV-positive men who have sex with men [90]. The app is centred on a superhero storyline, in which users battle to get points, complete daily information modules, and track their medication intake, among other features. Usability testing has shown the app to be received positively and users found the app engaging and relevant. Social aspects of the game were highlighted as a notable positive of the game, however, users desired greater interactivity in the game-based components of the app, such as battling. The app has been modified based on user feedback.

AllyQuest

AllyQuest is a gamified mobile app designed to increase antiretroviral medication adherence and social support and improve engagement in care in young, HIV-positive men who have sex with men [91]. Users participate in daily activities, choose-your-own adventure stories, discussions with other users, and are reinforced with virtual rewards and achievements. A pilot trial of AllyQuest found that higher levels of app usage (days logged in) were positively correlated with HIV self-management outcomes and confidence in one’s ability to take their medication reliably post-trial. Acceptability ratings for the app were high and the gamification aspects, medication tracker and daily discussion features were rated positively. Engagement with the app was acceptable (average 21.2 days over a four-week trial); however, usage declined over the course of the trial. The app has been refined based on pilot user feedback.

53. Lucassen, M. et al., *How LGBT+ young people use the internet in relation to their mental health and envisage the use of e-therapy: exploratory study.* JMIR Serious Games, 2018;6(4):e11249

64. Mustanski, B., et al., *Feasibility, acceptability, and initial efficacy of an online sexual health promotion program for LGBT youth: The Queer Sex Ed intervention.* The Journal of Sex Research, 2015. **52**(2): p. 220-230.

65. Martin, S., *Developing and evaluating QueerViBE: An online intervention to empower trans and non-binary youth*. 2019, University of Greenwich, London, UK.

66. Mitchell, J.T., et al., *Smartphone-Based Contingency Management Intervention to Improve Pre-Exposure Prophylaxis Adherence: Pilot Trial.* JMIR Mhealth Uhealth, 2018. **6**(9): p. e10456.

67. Greene, G.J., et al., *Implementation and Evaluation of the Keep It Up! Online HIV Prevention Intervention in a Community-Based Setting.* AIDS Education & Prevention, 2016. **28**(3): p. 231-45.

68. Madkins, K., et al., *Measuring Acceptability and Engagement of The Keep It Up! Internet-Based HIV Prevention Randomized Controlled Trial for Young Men Who Have Sex With Men.* AIDS Education & Prevention, 2019. **31**(4): p. 287-305.

69. Mustanski, B., et al., *Feasibility, acceptability, and preliminary efficacy of an online HIV prevention program for diverse young men who have sex with men: the keep it up! intervention.* AIDS and Behavior, 2013. **17**(9): p. 2999-3012.

70. Mustanski, B., et al., *Biomedical and Behavioral Outcomes of Keep It Up!: An eHealth HIV Prevention Program RCT.* American journal of preventive medicine, 2018.

71. Christensen, J.L., et al., *Reducing shame in a game that predicts HIV risk reduction for young adult MSM: a randomized trial delivered nationally over the Web.* Journal of the International AIDS Society, 2013. **16**(3 Suppl 2): p. 18716.

72. Hightow-Weidman, L., et al., *HealthMpowerment.org: development of a theory-based HIV/STI website for young black MSM.* AIDS Education & Prevention, 2011. **23**(1): p. 1-12.

73. Hightow-Weidman, L., et al., *A Randomized Trial of an Online Risk Reduction Intervention for Young Black MSM.* AIDS & Behavior, 2019. **23**(5): p. 1166-1177.

74. Hightow-Weidman, L., et al., *HealthMpowerment. org: feasibility and acceptability of delivering an internet intervention to young Black men who have sex with men.* AIDS care, 2012. **24**(7): p. 910-920.

75. Ybarra, M.L., et al., *Online focus groups as an HIV prevention program for gay, bisexual, and queer adolescent males.* AIDS Education and Prevention, 2014. **26**(6): p. 554-564.

76. Ybarra, M.L., et al., *The Effect of a Text Messaging Based HIV Prevention Program on Sexual Minority Male Youths: A National Evaluation of Information, Motivation and Behavioral Skills in a Randomized Controlled Trial of Guy2Guy.* AIDS and behavior, 2018: p. 1-10.

77. Ybarra, M.L., et al., *Feasibility, Acceptability, and Process Indicators for Guy2Guy, an mHealth HIV Prevention Program for Sexual Minority Adolescent Boys.* Journal of Adolescent Health, 2019.

78. Ybarra, M.L., et al., *Iteratively developing an mHealth HIV prevention program for sexual minority adolescent men.* AIDS and Behavior, 2016. **20**(6): p. 1157-1172.

79. Ybarra, M.L., et al., *Pilot RCT results of an mHealth HIV prevention program for sexual minority male adolescents.* Pediatrics, 2017. **140**(1): p. e20162999.

80. Solorio, R., et al., *Tu Amigo Pepe: Evaluation of a Multi-media Marketing Campaign that Targets Young Latino Immigrant MSM with HIV Testing Messages.* AIDS & Behavior, 2016. **20**(9): p. 1973-88.

81. Cho, H., et al., *A Mobile Health Intervention for HIV Prevention Among Racially and Ethnically Diverse Young Men: Usability Evaluation.* JMIR mHealth and uHealth, 2018. **6**(9): p. e11450.

82. Muessig, K.E., et al., *"I Didn't Tell You Sooner Because I Didn't Know How to Handle It Myself." Developing a Virtual Reality Program to Support Hiv-Status Disclosure Decisions.* Digital Culture & Education, 2018. **10**: p. 22-48.

83. Bauermeister, J.A., et al., *Acceptability and Preliminary Efficacy of an Online HIV Prevention Intervention for Single Young Men Who Have Sex with Men Seeking Partners Online: The myDEx Project.* AIDS & Behavior, 2019. **14**: p. 14.

84. Burnham, B.M., *Addressing the Epidemic of Anal Human Papillomavirus (HPV) Infection via an E-Health Video Designed to Empower Young Men Who Have Sex With Men (YMSM) to Increase HPV Screening and Vaccination, Screening for HIV and Other Sexually Transmitted Infections, and Adoption of Risk Reduction Behaviors*. 2017, Teachers College, Columbia University: Ann Arbor. p. 259.

85. McRee, A.L., et al., *Outsmart HPV: Acceptability and short-term effects of a web-based HPV vaccination intervention for young adult gay and bisexual men.* Vaccine, 2018. **36**(52): p. 8158-8164.

86. Bauermeister, J.A., et al., *Acceptability and preliminary efficacy of a tailored online HIV/STI testing intervention for young men who have sex with men: the Get Connected! program.* AIDS and Behavior, 2015. **19**(10): p. 1860-1874.

87. Horvath, K.J. and J.A. Bauermeister, *eHealth literacy and intervention tailoring impacts the acceptability of a HIV/STI testing intervention and sexual decision making among young gay and bisexual men.* AIDS Education and Prevention, 2017. **29**(1): p. 14-23.

88. Washington, T.A., S. Applewhite, and W. Glenn, *Using Facebook as a Platform to Direct Young Black Men Who Have Sex With Men to a Video-Based HIV Testing Intervention: A Feasibility Study.* Urban Social Work, 2017. **1**(1): p. 36-52.

89. McCoy, S.I., et al., *Stick To It: pilot study results of an intervention using gamification to increase HIV screening among young men who have sex with men in California.* Mhealth, 2018. **4**.

90. LeGrand, S., et al., *Epic Allies: development of a gaming app to improve antiretroviral therapy adherence among young HIV-positive men who have sex with men.* JMIR serious games, 2016. **4**(1): p. e6.

91. Hightow-Weidman, L., et al., *A Gamified Smartphone App to Support Engagement in Care and Medication Adherence for HIV-Positive Young Men Who Have Sex With Men (AllyQuest): Development and Pilot Study.* JMIR Public Health and Surveillance, 2018. **4**(2): p. e34.

92. Schwinn, T.M., et al., *Preventing drug use among sexual-minority youths: findings from a tailored, web-based intervention.* Journal of Adolescent Health, 2015. **56**(5): p. 571-573.

93. Lucassen, M., *Evaluation of a computerised cognitive behavioural therapy program for depressive symptoms in sexual minority youth*. 2012, ResearchSpace@ Auckland.

94. Lucassen, M., et al., *A qualitative study of sexual minority young people’s experiences of computerised therapy for depression.* Australasian Psychiatry, 2015. **23**(3): p. 268-273.

95. Lucassen, M., et al., *Rainbow SPARX: A novel approach to addressing depression in sexual minority youth.* Cognitive and Behavioral Practice, 2015. **22**(2): p. 203-216.

96. Fleming, J.B., Y.N. Hill, and M.N. Burns, *Usability of a culturally informed mHealth intervention for symptoms of anxiety and depression: feedback from young sexual minority men.* JMIR human factors, 2017. **4**(3): p. e22.

97. Pachankis, J.E. and M.R. Goldfried, *Expressive writing for gay-related stress: Psychosocial benefits and mechanisms underlying improvement.* Journal of Consulting and Clinical Psychology, 2010. **78**(1): p. 98.

98. Vogel, E.A., et al., *Development and acceptability testing of a Facebook smoking cessation intervention for sexual and gender minority young adults.* Internet Interventions, 2019.

99. Vogel, E.A., et al., *Smoking cessation intervention trial outcomes for sexual and gender minority young adults.* Health Psychology, 2019. **38**(1): p. 12.

100. Merry, S.N et al., *The effectiveness of SPARX, a computerised self help intervention for adolescents seeking help for depression: randomised controlled non-inferiority trial*. Br Med J, 2012.

101. Reiter ,P.L. et al., *Increasing human papillomavirus vaccination among young gay and bisexual men: a randomized pilot trial of the outsmart HPV intervention*. LGBT Health, 2018. p. 325-329
